# Germination Dynamics and Seedling Development of Wheat Under Various Ionic Salt Stresses

**DOI:** 10.3390/plants15111671

**Published:** 2026-05-29

**Authors:** Indrila Dey Traye, Nur Mohammod Oli, Hongyu Zheng, Kangjun Wang, Yijun Shi, Tianyao Meng, Guanglong Zhu, Guisheng Zhou, Yunji Xu

**Affiliations:** 1 Joint International Research Laboratory of Agriculture and Agri-Product Safety, The Ministry of Education of China, Yangzhou University, Yangzhou 225009, China; indriladeytraye@gmail.com (I.D.T.); zhenghongyu_1027@126.com (H.Z.); tymeng@yzu.edu.cn (T.M.); g.zhu@yzu.edu.cn (G.Z.); 2 College for Overseas Education, Yangzhou University, Yangzhou 225009, China; hasanoly9@gmail.com; 3 Lianyungang Academy of Agricultural Sciences, Lianyungang 222000, China; kjwang13@163.com (K.W.); shiyijunhappy@163.com (Y.S.)

**Keywords:** wheat, salt type, salt stress, germination, seedling growth

## Abstract

A significant abiotic stressor that negatively impacts plant seed germination and seedling establishment is soil salinization, especially in staple crops like wheat (*Triticum aestivum* L.). The complex ionic stressors that make up salinity include divalent salts (MgCl_2_), alkaline salts (NaHCO_3_), and neutral salts (NaCl, KCl), each of which has unique effects on osmotic and ionic toxicity. The present understanding of how various ionic salt stressors affect the dynamics of wheat germination and the early development of seedlings is summarized in this article. We talk about physiological and biochemical reactions, possible adaptive mechanisms, and the ionic specificity of toxicity. Important research findings show that: (1) germination rate and seedling vigor are reduced in response to salt content; (2) growth parameters are affected by ionic composition; and (3) genotypic variability in salt sensitivity is observed in response to salinity stress. Improving wheat performance in saline soils and developing breeding plans for salt tolerance require an understanding of these dynamics.

## 1. Introduction

Wheat (*Triticum aestivum* L.) is a major source of protein and calories for people all over the world. Although wheat is categorized as a crop that can endure some salt, its yield is extremely susceptible to salinity, especially in the early phases of development [1,2]. Since they affect plant population density, early vigor, and the potential for further growth and yield formation, seed germination and seedling establishment are thought to be the critical salt-sensitive stages [3]. There are two main ways that salt stress impacts plant growth: osmotic stress and ionic stress. Immediately following exposure to salt, osmotic stress reduces seeds’ absorption of water and delays imbibition and metabolic activation [4]. When too many ions build up in plant tissues, cellular homeostasis, enzyme activity, and membrane stability are all disrupted, leading to ionic stress [5]. Reactive oxygen species (ROS) overproduction frequently results in secondary oxidative stress, which worsens cellular structures and physiological functions [6]. Wheat seeds are especially susceptible to oxidative damage caused by salt because of their low antioxidant capacity during germination and the early stages of seedling growth [7].

Early wheat development is severely hampered by salinity, which significantly reduces biomass accumulation, root and shoot elongation, and overall seedling vigor. Root tissues are particularly vulnerable because of their direct exposure to saline substrates and quick ion absorption dynamics [8]. During the early stages of development, excessive absorption of harmful ions, especially Na^+^, interferes with mitotic activity, inhibits cell elongation, and destabilizes membrane integrity, all of which affect cellular homeostasis. Protein biosynthesis, photosynthetic efficiency, and energy metabolism are all hampered by the early buildup of Na^+^ because it upsets the intracellular Na^+^/K^+^ balance, which is a crucial factor in enzyme control, osmotic adjustment, and metabolic activity [9]. When divalent and alkaline salts predominate, these negative effects become significantly more noticeable. Ionic toxicity is exacerbated by increased pH stress and worsened nutritional imbalance, which together lead to sharp declines in seedling viability and survival [10]. The reactions of wheat genotypes differ greatly: resistant lines retain superior germination, root growth, and ion balance in comparison to sensitive ones [11]. These adaptive characteristics help maintain metabolic activity in saline environments, improve osmotic management, and lessen oxidative damage. Given that seedling performance under salinity is often linked to later developmental resilience and yield stability in salt-affected environments, this heterogeneity highlights the crucial significance of early-stage phenotypic screening in breeding efforts [12]. However, genotypes adapted to more complicated, mixed-salt environments may be overlooked if breeding programs just rely on NaCl.

The majority of conventional research on wheat’s ability to endure salt uses NaCl as the main stressor. Natural saline soils, on the other hand, are far more complex and include a variety of salts, including KCl, NaCl, NaHCO_3_ and MgCl_2_ [13,14]. These salts cause different physiological and biochemical reactions in plants due to differences in their ionic composition and chemical behavior. As a result, NaCl by itself may underestimate the influence of other ions and oversimplify plant stress responses [15,16]. There is growing evidence that the type of salt greatly affects wheat germination characteristics, including uniformity, pace, and percentage. Alkaline salts, such as NaHCO_3_, increase toxicity and decrease nutritional availability by raising pH and intensifying osmotic and ionic stress [17]. By interfering with calcium signaling and membrane integrity, divalent cations like Mg^2+^ worsen stress [18]. Potassium’s crucial involvement in osmotic equilibrium and enzyme function is highlighted by the fact that KCl tends to be less inhibitory [19].

Despite tremendous advancements in our knowledge of wheat salinity tolerance at the physiological, biochemical, and molecular levels, there are still few thorough reviews that concentrate on the effects of ionic salts on germination and seedling development. The majority of evaluations that are now available focus on whole-plant reactions, ion transport systems, or yield-related characteristics under salinity; the early phases of development and the significance of various salt types receive comparatively less attention [20,21]. There is an urgent need for a targeted synthesis of current information in this field because germination and early seedling growth are crucial for crop establishment. Thus, the objective of this review is to critically assess and methodically analyze the effects of different ionic salt stresses on wheat germination dynamics and seedling growth. The differences between neutral, alkaline, and divalent salts, the physiological and biochemical mechanisms behind ionic toxicity during early growth stages, and the consequences of these results for wheat breeding programs and salt tolerance screening are all given special attention. This review aims to support the creation of more practical evaluation techniques and better management of wheat production in salt-affected soils by offering an integrated overview of ion-specific responses.

## 2. Impacts of Ionic Salt Stress on Wheat Germination and Seedling Development

### 2.1. Effects of Salt Type and Concentration on Seed Germination

**Figure 1:**
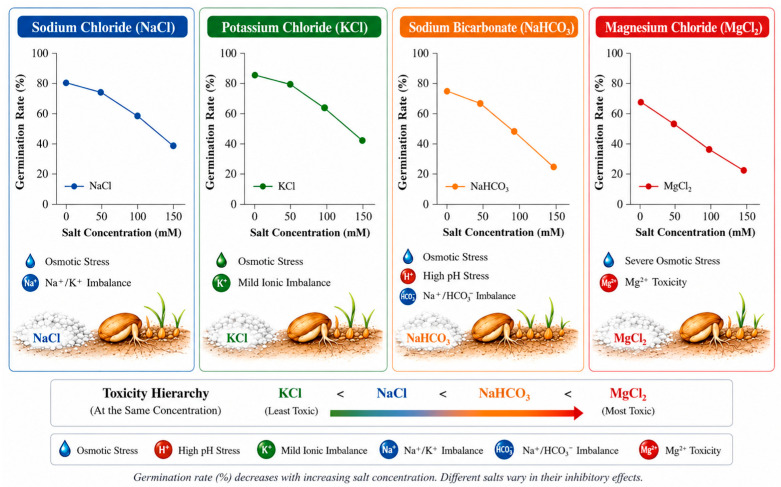
Impact of salt type and concentration on wheat germination (graphing with Adobe Illustrator, Bio Render, Canva).

The physiological response of wheat during germination and early ontogeny is highly dependent on both ionic composition and salinity intensity because natural edaphic environments are characterized by heterogeneous assemblages of neutral, alkaline, and divalent salts. As salt concentration rises, germination kinetics, seedling vigor, and emergence percentage are subject to progressively harsher restrictions, indicating a concentration-dependent disturbance of cellular homeostasis. Low to moderate salinity mainly causes osmotic stress, which lowers external water potential, hinders imbibition, and delays radicle protrusion, as shown in Figure 1 [20,21]. Ionic toxicity takes over as the primary inhibitory force in high salinity regimes, causing irreversible metabolic disruptions, membrane instability, and cytotoxic damage. According to experimental data, wheat seeds have a limited ability to withstand mildly saline conditions, frequently showing only temporary germination delays; however, exposure to high salt concentrations significantly inhibits the activity of hydrolytic enzymes linked to endosperm reserve mobilization, especially α-amylase, thereby limiting the availability of carbohydrates for embryonic development [4]. Additionally, excessive ionic buildup increases the production of reactive oxygen species (ROS) and upsets the balance of phytohormones, both of which worsen oxidative damage, interfere with cellular communication pathways, and lower germination synchrony and seedling establishment efficiency [22]. Therefore, salt concentration and ionic specificity are inextricably connected to the extent, duration, and reversibility of salinity-induced damage throughout the early stages of development. Neutral salts like sodium chloride and potassium chloride continue to be the most researched salinizing agents because of their widespread occurrence and significant impact on wheat germination dynamics, as seen in Figure 1. Generally, osmotic stress at low concentrations and Na^+^ toxicity at higher concentrations are the main ways that NaCl inhibits wheat germination in a concentration-dependent manner [13]. Enzyme activity and membrane stability are hampered by excess Na^+^, which upsets the Na^+^/K^+^ balance. At comparable osmotic potentials, however, KCl frequently exhibits weaker inhibitory effects than NaCl. Since potassium is a necessary macronutrient for stomatal function, osmoregulation, and enzyme activation, a modest dose of K^+^ may help reduce osmotic stress during germination [23]. However, due to its high ionic strength, KCl also has harmful effects at high doses, decreasing germination and seedling growth [24].

In spite of the combined combination of osmotic stress, ionic toxicity, and alkaline pH-mediated physiological disturbance, alkaline salts like sodium bicarbonate impose a complex form of salinity stress that is far more harmful than that caused by neutral salts. Elevated alkalinity significantly changes rhizospheric chemical equilibria in contrast to neutral saline conditions, which lowers the bioavailability and uptake efficiency of vital macronutrients and micronutrients, such as Fe, Zn, and P, which are critical for enzymatic activation and embryonic metabolism during germination [17]. Research continuously shows that at comparable concentrations, alkaline salts limit wheat germination more than NaCl. Even at relatively low concentrations, NaHCO_3_ strongly slows early seedling growth, lowers germination energy, and delays the commencement of germination [14]. Alkaline salinity is especially harmful to wheat establishment during the crucial early stages of development because of the synergistic interaction between excessive Na^+^ accumulation and high pH stress, which exacerbates membrane instability, disturbs intracellular ion homeostasis, and intensifies oxidative injury. The most potent inhibitors of wheat germination and seedling development are frequently divalent salts like magnesium chloride (MgCl_2_). Mg^2+^ ions cause cell membrane instability, interfere with Ca^2+^ uptake and signaling, and interfere with enzyme activity [18]. Consequently, with Mg^2+^-dominated salinity, germination percentage and seedling vigor drastically decrease. Ionic composition is more important than total salinity alone, as evidenced by comparative studies showing that MgCl_2_ frequently shows more toxicity than NaCl at equal electrical conductivity levels [10]. The limits of NaCl-based screening devices are highlighted by the various effects of salt type and concentration. Without taking ionic composition into account, it is impossible to forecast how wheat will react to salt, especially in saline-alkaline and magnesium-rich soils. The dependability of breeding programs and assessments of salt tolerance will be enhanced by incorporating a variety of salt types and concentration gradients into germination experiments [25]. Tolerance screening is more reliable when a variety of salts and concentration gradients are used. Figure 1 shows the overall trend of salt toxicity is KCl < NaCl < NaHCO_3_ < MgCl_2_, highlighting the crucial role that ionic composition plays in determining wheat germination responses.

### 2.2. Mechanisms of Inhibition of Wheat Germination Under Ionic Salt Stress

**Figure 2:**
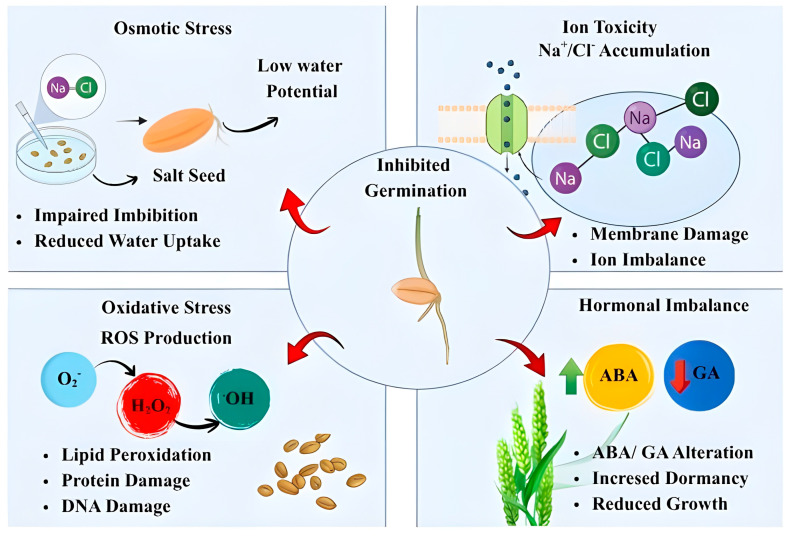
Mechanisms of inhibition of wheat germination under ionic salt stress (graphing with Adobe Illustrator, Bio Render, Canva; the upward green arrow indicates an increase in ABA, while the downward red arrow represents a decrease in GA in the figure).

Water intake, metabolic activation, enzymatic hydrolysis, and controlled cellular proliferation are all precisely regulated during the highly coordinated developmental process of seed germination. Osmotic stress, ionic toxicity, secondary oxidative damage, and hormonal disequilibrium are the four main stress components that interfere with this physiological equilibrium in saline environments, as shown in Figure 2. This leads to delayed emergence, a lower germination percentage, and poor seedling uniformity [26,27]. Osmotic stress is the first and most direct reaction to exposure to salinity among these limitations. High quantities of soluble salts significantly reduce the germination medium’s external osmotic potential, creating a situation known as “physiological drought,” where water is still physically present but is no longer osmotically accessible to the seed [28]. As a result, imbibition—the crucial first stage of germination—is severely limited, which reduces the amount of cellular water needed for metabolic reactivation. Key biochemical processes involved in reserve mobilization and embryonic development are suppressed by inadequate water uptake, especially the activity of hydrolytic enzymes like α-amylase, which catalyzes the conversion of stored starch reserves into soluble sugars required for respiration and energy production [29]. Important processes including radicle emergence, membrane loosening, and protein synthesis therefore do not take place. Thus, osmotic stress alone can effectively mimic dry conditions and significantly impede germination even in water-rich soils [30].

Certain ions in saline settings directly interfere with cellular structure and metabolism, going beyond osmotic effects. Excessive Na^+^ and Cl^−^ accumulation causes ionic toxicity, which damages membranes and upsets nutritional balance, as seen in Figure 2 [31]. High Na^+^ disrupts the Na^+^/K^+^ ratio and hinders protein synthesis and metabolic processes by competing with K^+^, an essential ion for enzyme activation and osmotic regulation [32]. Increased Cl^−^ further disrupts membrane potential and cellular pH, limiting enzyme function and nutritional absorption. As a secondary effect of ionic and osmotic imbalance, salt stress also causes oxidative damage [33]. Reactive oxygen species (ROS) including O_2_^−^·, H_2_O_2_, and ·OH are produced in excess as a result, overwhelming antioxidant defenses such superoxide dismutase, catalase, and peroxidases [34]. Membrane stability and metabolic function are compromised by the ensuing damage, which includes lipid peroxidation, protein degradation, and DNA disruption. Long-term salt affects seeds’ ROS-scavenging mechanisms, which ultimately lowers germination success and seedling vigor [35].

By affecting important phytohormones, salt stress modifies the hormonal control of seed germination. A hormone called abscisic acid (ABA), which encourages dormancy, is frequently elevated in saline environments, which strengthens dormancy and prevents the start of germination (Figure 2). Conversely, hormones that promote germination, such as gibberellins (GA), cytokinins, and ethylene, often decrease or become dysregulated, further suppressing metabolic activation and embryo growth [36]. Particularly important is the ABA/GA balance. In promoting abscisic acid (ABA) biosynthesis and inhibiting gibberellin (GA)-mediated growth pathways, high salinity upsets the hormonal balance during seed germination, resulting in circumstances that prevent germination and early seedling establishment. Furthermore, osmotic stress and dehydration reactions are triggered by salinity and are controlled by ABA-dependent signaling pathways. Transcription factors including AREB/ABF, bZIP, and DREB, which control downstream stress-responsive genes linked to osmoprotection, antioxidant defense, and seed dormancy, are activated by increased ABA accumulation in saline environments. Additionally, recent research indicates that salt stress dramatically induces genes involved in ABA production, such as those in the NCED family, which contributes to the high ABA/GA ratio that inhibits germination and early growth [37]. Even in situations where other factors (such as temperature, oxygen, and light) are favorable, the general result is a delayed or diminished germination response, even though particular molecular triggers differ by species [38]. The combined effects of ionic imbalance, metabolic disruption, and salinity-induced osmotic constraints in wheat result in significant declines in seedling vigor indices, longer germination times, and lower final germination percentages—all of which are crucial for successful stand establishment in saline soil. Ionic composition and ion-specific interactions within cellular systems have a significant impact on the intensity of these inhibitory responses in addition to concentration. Specifically, compared to monovalent ions linked to salts like NaCl, divalent cations like Mg^2+^ show higher electrostatic interactions with membrane phospholipids, transport proteins, and nutrition acquisition pathways. These interactions can exacerbate metabolic dysfunction during germination and early seedling growth by increasing membrane stiffness, interfering with the uptake and translocation of vital nutrients, and disrupting selective ion transport. As a result, the physicochemical characteristics and biological reactivity of the main ionic constituents as well as the overall salt concentration determine the phytotoxicity of saline environments in wheat [39].

### 2.3. Effects of Ionic Salt Stress on Seedling Development

#### 2.3.1. Early Growth Responses

**Figure 3:**
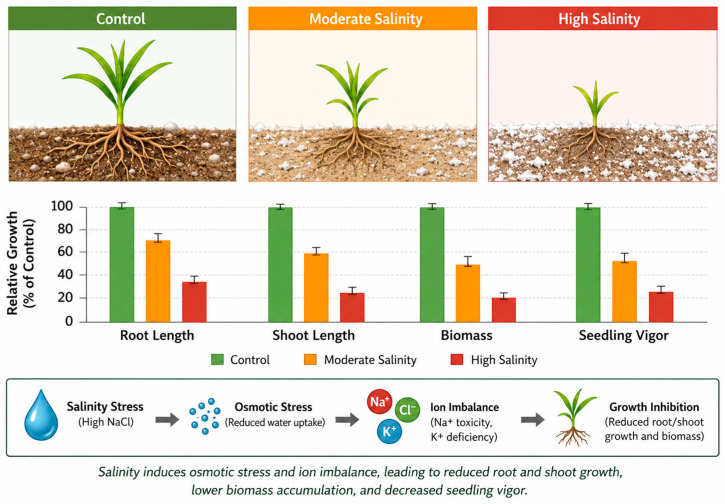
Wheat seedling response to salinity stress (graphing with Adobe Illustrator, Bio Render, Canva).

The transition from germination to autotrophic development is represented by early seedling growth, which depends on effective reserve mobilization, rapid cell division, and elongation (Figure 3). These processes are significantly changed by ionic salt stress, which has a significant impact on biomass accumulation, root length, shoot length, and seedling vigor [40]. The degree of inhibition is highly influenced by the type and concentration of salt. In wheat seedlings, root development is typically the most sensitive marker of early salt stress. The direct exposure of roots to saline media causes ion uptake and buildup to occur quickly. Increasing salinity dramatically decreases main root elongation and lateral root growth, according to numerous research [21,41]. Shorter and thicker roots are the result of excessive Na^+^ and Cl^−^ disrupting cell division in the root meristem and reducing cell wall extensibility. By disrupting Ca^2+^ uptake and membrane stability, which are critical for signal transmission and root tip integrity, divalent salts like MgCl_2_ have very potent inhibitory effects on root growth [18]. By increasing the pH of the rhizosphere, decreasing the solubility of nutrients, and inhibiting the metabolic activity of roots, alkaline salts exacerbate the suppression of root growth [17]. Even while roots are the main location where salinity is perceived in the early stages of ontogenesis, salt stress also has a significant inhibitory influence on the development of shoots, even though shoots are typically more tolerant in the early stages of growth. Impaired cellular expansion, decreased transfer of assimilates from endosperm stores, and disruption of phytohormonal signaling networks that control vegetative development are the main causes of the inhibition of shoot elongation under saline conditions [20]. High salt concentrations restrict the growth of both fresh and dry matter in growing seedlings by interfering with basic metabolic processes such as protein production, enzyme activity, and energy consumption. Hormonal imbalances brought on by salinity, especially those involving gibberellins, auxins, and abscisic acid, further restrict meristematic activity and prevent shoot differentiation and elongation. It is interesting to note that some wheat genotypes may show temporary stimulation of shoot growth under mild saline conditions due to osmotic adjustment mechanisms or increased K^+^ availability, particularly when KCl treatments are present [23]. This transient adaptive reaction probably results from better ionic equilibrium and cellular turgor maintenance. However, the positive effects of ionic adjustment are quickly overshadowed by severe metabolic inhibition, ion toxicity, and growth suppression as salinity intensity rises above the physiological tolerance threshold. This ultimately leads to noticeable declines in shoot biomass and overall seedling performance.

The root-to-shoot ratio is frequently changed by salt stress, indicating varying organ sensitivity. A lower root-to-shoot ratio and a weakened ability to absorb water and nutrients result from the fact that shoot growth is frequently less hindered than root growth (Figure 3) [9]. Such morphological instability significantly limits the growth of following plants and jeopardizes the stability and establishment of seedlings in saline environments. Changes in root-to-shoot architecture brought on by salinity impair the coordinated distribution of resources needed for ideal growth, which lowers seedlings’ ability to maintain effective water uptake, nutrient transport, and photosynthetic activity. Some salt-tolerant wheat genotypes show adaptive morphological changes in response to these unfavorable conditions, such as increased root thickness, decreased leaf surface area, and altered tissue succulence. These changes may help minimize excessive ion uptake and limit transpirational water loss. By enhancing osmotic regulation and lowering exposure to ionic toxicity, these structural modifications improve survival; yet, they are often accompanied by notable trade-offs in growth performance. Even in genotypes with relatively high saline tolerance, the diversion of metabolic energy into stress adaptation and maintenance activities frequently leads to decreased biomass formation, limited vegetative development, and decreased overall production [26]. Under ionic salt stress, the seedling vigor index (SVI), which combines the germination percentage with the seedling length, drastically decreases. By concurrently preventing early development and germination speed, high salinity lowers SVI and produces weak, uneven seedlings [3]. In saline settings, poor seedling vigor is closely associated with decreased field establishment and yield potential. Alkaline and divalent salts produce significant growth suppression and low survival rates, while neutral salts often result in mild reductions in seedling vigor, according to comparative studies [10]. These results highlight the use of early development features as trustworthy markers for determining a wheat genotype’s capacity to withstand salt. Wheat salt tolerance mechanisms can be better understood by examining early development responses. Under controlled circumstances, traits including biomass accumulation, root-to-shoot ratio, shoot length, and root length are frequently employed for quick screening. The accuracy of tolerance testing is increased and the applicability of laboratory results to field circumstances is improved by using different salt kinds and concentration gradients [12].

#### 2.3.2. Ionic and Physiological Mechanisms

An intricate array of ionic and physiological abnormalities brought on by salt stress during the early stages of wheat seedling development hinder growth, metabolism, and establishment. The severity of these mechanisms, which are largely caused by ion toxicity, nutritional imbalance, membrane dysfunction, and disruption of physiological processes, is dependent on the concentration and composition of salt [3]. The excessive buildup of harmful ions, especially Na^+^ and Cl^−^, in plant tissues is one of the most significant consequences of salinity on wheat seedlings. The plant’s ability to compartmentalize or exclude sodium is overpowered by high external Na^+^ concentrations, which encourage passive influx into root cells [20]. K^+^, a crucial macronutrient needed for protein synthesis, osmotic control, and enzyme activation, is in competition with excess Na^+^. Consequently, under salt, the Na^+^/K^+^ ratio rises dramatically, causing growth inhibition and metabolic dysfunction [42]. Cl^−^ accumulation limits nitrogen metabolism during early growth by interfering with nitrate uptake and disrupting cellular electroneutrality, in addition to Na^+^ toxicity [43]. Divalent ions like Mg^2+^, which are frequently found in saline soils as MgCl_2_, worsen ionic stress by obstructing the uptake of Ca^2+^, which is necessary for intracellular signaling and membrane stability [18].

During seedling establishment, maintaining membrane integrity is essential for regular physiological function. High concentrations of Na^+^ and Mg^2+^ destabilize plasma membranes and tonoplasts under ionic salt stress by dislodging membrane-bound Ca^2+^, which increases membrane permeability and electrolyte leakage [9]. Selective ion uptake and cellular compartmentalization are hampered by this membrane damage, which interferes with ion transport systems such as H-ATPases and ion channels. Additionally, the sequestration of toxic ions into vacuoles is limited when membrane-associated transporters are damaged, which raises their cytosolic concentrations and intensifies toxicity [21]. All of these factors work together to make young wheat seedlings less efficient at acquiring nutrients and coordinating their metabolism. Changes in plant water relations are another physiological reaction to salinity. Excessive levels of salt reduce the external water potential, which lowers water uptake and dehydrates cells. Proline, soluble sugars, and organic acids are among the compatible solutes that wheat seedlings accumulate in order to counteract this and start the osmotic adjustment process [44]. Osmotic adjustment takes a lot of metabolic energy and takes resources away from growth processes, even though it aids in maintaining turgor pressure. Reduced cell expansion and poor root and shoot elongation are caused by inadequate osmotic adjustment in salt-sensitive genotypes. On the other hand, during early growth, salt-tolerant genotypes typically show better water status maintenance and more effective solute accumulation [21]. During early development, wheat seedlings are still partially heterotrophic, but as leaves emerge, photosynthetic machinery is quickly impacted by ionic stress brought on by salinity. Elevated Na^+^ concentrations decrease photosystem II efficiency, interfere with thylakoid membrane structure, and hinder chlorophyll synthesis [45]. Growth of seedlings is further restricted by reduced photosynthetic capacity, which limits the availability of carbohydrates. By reducing mitochondrial enzyme activity and changing energy production, salinity also has an impact on respiratory metabolism. The availability of ATP, which is necessary for active transport, biosynthesis, and cell division in the early stages of seedling development, is decreased by ion-induced metabolic imbalance [46]. Under salinity stress, ionic toxicity and physiological impairment are inextricably linked. Ion disequilibrium causes membrane instability, which exacerbates disturbances in ion transport and cellular water balance through a self-amplifying stress cascade. Therefore, the ability to maintain minimal cytosolic Na^+^ buildup, maintain appropriate Na^+^/K^+^ balance, sustain osmotic adjustment, and safeguard membrane structural integrity is the primary factor governing wheat’s early-stage salt tolerance [47]. A crucial basis for the creation of improved screening techniques and wheat genotypes resistant to salinity is provided by the clarification of these linked physiological and ionic mechanisms.

## 3. Screening Wheat Germplasm for Salt Tolerance

Crop improvement is based on genetic variability, and wheat genotypes differ significantly in their ability to germinate, establish, and survive in saline conditions [20]. Because poor germination and weak seedling establishment directly affect stand density and production, early-stage screening has emerged as a useful and economical method for finding salt-tolerant genotypes [48]. Considering their ease of use and good correlation with salinity performance, morphological characteristics such as germination percentage, germination rate, root and shoot length, seedling biomass, and seedling vigor index (SVI) are still often used. Under stress, salt-tolerant genotypes typically show better seedling establishment and less growth inhibition [21]. Because root characteristics immediately reflect salt exposure, water intake, and ion control, they are very instructive [49]. However, morphological characteristics by themselves are unable to distinguish between real ionic tolerance and osmotic adaptation. As a result, when combined with physiological and biochemical markers like relative water content (RWC), membrane stability index (MSI), chlorophyll retention, electrolyte leakage, and maintenance of Na^+^/K^+^ homeostasis, which all offer a deeper understanding of stress acclimation mechanisms, their interpretive value is significantly increased.

One important indicator of wheat salt tolerance is a low Na^+^/K^+^ ratio, which indicates efficient sodium exclusion or compartmentalization [42]. Additionally, salt-tolerant genotypes typically exhibit improved cellular structure protection under ionic stress, reduced electrolyte leakage, and increased membrane stability [50]. Furthermore, they frequently retain greater levels of chlorophyll and photosynthetic capacity, which enables them to continue growing in conditions of mild salinity [45]. The use of biochemical markers to enhance salt tolerance screening is growing. Antioxidant enzyme activity (SOD, CAT, POD), proline buildup, soluble sugar content, and lipid peroxidation (MDA levels) are significant markers. Genotypes with stronger antioxidant defenses typically experience less cellular damage and show better tolerance because salinity frequently causes oxidative stress through reactive oxygen species (ROS) [51]. Additionally, proline accumulation is well known for its function in ROS detoxification, protein stability, and osmotic correction [44]. Biochemical markers, however, need to be read cautiously because elevated metabolite levels can indicate either severe stress injury or adaptive defense. Salt tolerance screening has been further enhanced by developments in molecular biology, which have made it possible to combine classic physiological and biochemical methods with genetic and genomic tools.

In wheat, candidate genes including the HKT, NHX, and SOS families—which are linked to ion transport and stress signaling—are extensively researched [21]. While vacuolar antiporters like NHX1 compartmentalize excess Na^+^ into vacuoles to lessen cytosolic toxicity, the SOS1 plasma membrane transporter promotes Na^+^ extrusion from cells [5]. Similarly, HKT1;5 prevents excessive sodium accumulation in shoots and young seedlings by aiding in the retrieval of sodium from xylem tissues [46]. Breeding efficiency is increased by identifying genomic areas associated with salinity tolerance features through the use of marker-assisted selection (MAS) and quantitative trait locus (QTL) mapping [20]. Gene networks controlling osmotic adjustment, antioxidant activity, and ion homeostasis are also revealed by transcriptomic and proteomic investigations [52]. Because gene expression is frequently environment-dependent, molecular techniques, notwithstanding their accuracy, supplement rather than replace phenotypic screening [53]. The most accurate evaluation of salt tolerance is offered by integrated screening methods that combine morphological, physiological, biochemical, and molecular characteristics due to the complexity of salinity stress [54]. Principal component analysis (PCA) and stress tolerance indices are examples of multivariate statistical methods that improve genotype discrimination and lessen trait redundancy. Additionally, as various ions impose different toxicity pathways, screening under numerous salt types (such as NaCl, KCl, NaHCO_3_, and MgCl_2_) improves ecological relevance [10,55]. The development of salt-tolerant wheat cultivars that can sustain production in saline soils is accelerated by effective germplasm screening. Finding characteristics that are consistently associated with field performance is still a top priority. To improve breeding results and selection accuracy, future work should incorporate field validation, controlled-environment testing, and molecular diagnostics [41,56].

Salt-tolerant wheat genotypes frequently exhibit increased expression of these genes, suggesting their significance in lowering oxidative damage during germination and seedling establishment [52]. By stabilizing proteins and cellular membranes under ionic and osmotic stress conditions, heat shock proteins (HSPs), late embryogenesis abundant (LEA) proteins, and dehydrins also aid in stress defense [57]. Furthermore, transcriptome research has revealed a number of transcription factor families implicated in salinity tolerance, such as NAC, WRKY, MYB, and bHLH proteins, which govern stress-responsive pathways governing growth regulation, ion transport, and metabolism [58]. The ionic makeup of the stress environment frequently causes these highly interconnected regulatory networks to change. For instance, divalent salts like MgCl_2_ may selectively impact calcium signaling and membrane-associated transport proteins, while alkaline salts like NaHCO_3_ cause ionic toxicity and modify pH-responsive gene expression [59,60].

## 4. Conclusions

The kind and number of salts in the environment have a significant impact on how wheat reacts to salt stress during germination and early seedling growth. This paper emphasizes that different salts, like NaCl, NaHCO_3_, MgCl_2_, and MgSO_4_, have diverse impacts on seed hydration, metabolic activity, ion balance, and early plant establishment; hence, salinity should not be viewed as a homogeneous stress factor. Among them, alkaline and divalent salts frequently have more potent inhibitory effects than neutral salts, highlighting the significance of assessing ion-specific toxicity instead of depending just on total salinity levels. Early developmental characteristics, including germination behavior, root elongation, shoot growth, and seedling vigor, offer important information about wheat’s ability to adjust to salinity. Internal physiological and biochemical changes, such as osmotic control, antioxidant defense, and ion transport efficiency, are intimately related to these outward reactions. When combined, these characteristics provide a useful foundation for recognizing tolerant germplasm and comprehending the processes underlying early-stage resilience. One of the main implications of recent research is that wheat salt tolerance screening should use more realistic salt combinations that reflect field circumstances rather than just single-salt laboratory models. This change will strengthen the selection of genotypes appropriate for agriculture in naturally saline and sodic soils and increase the significance of experimental results. Additionally, a more dependable framework for breeding advancement will be provided by combining traditional screening qualities with physiological markers and molecular techniques.

In the end, a better understanding of how ionic stress impacts the early phases of plant growth is necessary to improve wheat performance under salt. In addition to promoting the development of salt-tolerant cultivars, expanding this understanding can help manage saline soils more successfully and produce wheat sustainably in locations that are prone to stress.

## Data Availability

No new data were created or analyzed in this publication.
